# Munson’s Sign: An Obvious Finding to Explain Acute Vision Loss

**DOI:** 10.5811/cpcem.2019.5.42793

**Published:** 2019-07-08

**Authors:** Jake Gold, Vijai Chauhan, Siripong Rojanasthien, Jennifer Fitzgerald

**Affiliations:** * Saint Louis University, Department of Emergency Medicine, St. Louis, Missouri; † Saint Louis University Eye Institute, Department of Ophthalmology, St. Louis, Missouri

## Abstract

Keratoconus is a progressive disorder affecting the cornea, which causes the cornea to become weakened and conical in appearance. The resultant decrease in structural integrity of the cornea predisposes affected individuals to acute corneal hydrops, a break in Descemet’s membrane, the deepest layer of the cornea, resulting in pain and acute vision loss. We present here a case of this little-known cause of acute vision loss, and an example of Munson’s sign, which is a v-shaped protrusion of the lower eyelid on downward gaze that is characteristic of advanced keratoconus. We hope to highlight Munson’s sign as a simple identifier of keratoconus in an otherwise undiagnosed individual suspected of having acute corneal hydrops.

## CASE PRESENTATION

**Image 1:**
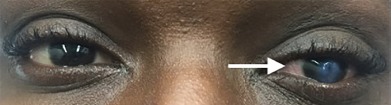
Obvious opacification of patient’s left cornea (arrow).

**Image 2:**
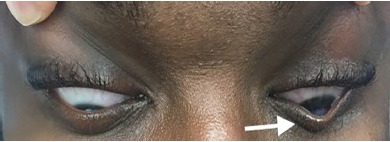
Positive Munson’s sign of patient’s left eye. Note the V-shaped protrusion of the left lower eyelid as the patient gazes downward (arrow).

A 28-year-old male with a history of keratoconus was transferred to our emergency department from an outside hospital for possible corneal ulcer. The patient suddenly lost vision in his left eye the night prior to presentation and had associated left eye pain and epiphora (watery eyes). The physical exam performed by ophthalmology noted opacification of the left cornea (Image 1) with a positive Munson’s sign (Image 2), central corneal edema, and oculus sinister (“left eye”) visual acuity decreased to hand motion at one foot. The remainder of the complete ophthalmologic exam revealed no additional abnormalities. The patient was diagnosed with acute corneal hydrops (ACH). He was given an eye shield without patching, started on cyclopentolate drops, erythromycin ointment, sodium chloride 5% drops, brimonidine drops, and instructed to avoid rubbing his eyes and to follow up with an ophthalmologist in one week.

## DISCUSSION

Keratoconus is a non-inflammatory disease characterized by progressive stromal thinning and cone-like bulging (ectasia) of the cornea leading to vision loss.^1^ It is the most common corneal ectatic disorder (CED).^1^ ACH, a complication of CEDs, is a break in Descemet’s membrane and the endothelium, the deepest layers of the cornea, resulting in corneal edema and a sudden, painful decrease in visual acuity.^2^ When ACH is suspected but there is no history of CED, a positive Munson’s sign can be used to make a diagnosis of keratoconus and subsequently ACH.^3^ Munson’s sign is pathognomonic for keratoconus.^3^ However, providers should be aware that Munson’s sign is typically seen in cases of advanced keratoconus, and its absence does not exclude CED or ACH.^3^

**CPC-EM Capsule**

What do we already know about this clinical entity?

Acute corneal hydrops (ACH) is a complication of keratoconus and other corneal ectatic disorders. Munson’s sign signifies advanced keratoconus.

What is the major impact of the image(s)?

The images presented demonstrate a couple of the simple ophthalmologic exam findings, particularly Munson’s sign, that can help emergency physicians identify keratoconus and ACH.

How might this improve emergency medicine practice?

*When present, Munson’s sign can help easily identify an individual with keratoconus without an ophthalmology consult.3*
